# Some Remarks upon the Use of the Forceps in Midwifery, Suggested on Reading a Paper on That Subject, Signed L. G., Contained in the Medical and Physical Journal for September

**Published:** 1820-10

**Authors:** A. B. Granville

**Affiliations:** Physician-Accoucheur to the Westminster General Dispensary; Principal Physician to the Royal Infirmary for the Diseases of Children; and Physician-in-ordinary to his Royal Highness the Duke of Clarence.


FOR THE LONDON MEDICAL AND PHYSICAL JOURNAL.

*Some Remarks upon the Use of the Forceps in Midwifery, suggested on reading a Paper on that subject, signed L. G., contained in the Medical and Physical Journal for September.* By A. B. GRANVILLE, M.D. F.R.S. &c. Physician-Accoucheur to the Westminster General Dispensary; Principal Physician to the Royal Infirmary for the Diseases of Children; and Physician-in-ordinary to his Royal Highness the Duke of Clarence.

THE paper upon which I am about to comment is highly creditable to the author, not only on account of the importance of its subject, but also from the very able manner in which that subject is treated. Papers of this nature, and of so much practical utility, are calculated to do more good than long and tedious treatises on Midwifery, wherein the unexperienced beginner looks in vain for directions to guide him through the intricate and perplexing difficulties of his art. To lay down any precise, clear, and undeviating practical rules, by which the junior practitioners in midwifery may, with safety to the patient, and credit as well as satisfaction to themselves, get over the anxious moment of instrumental application, when imperiously called for, is to confer on them a great obligation. This debt then, I have no hesitation in saying, the novices in midwifery owe to L. G. who, if discrimination fails not, appears to be a person of no ordinary stamp in the profession. Well may he assert, that such rules or instructions as he has detailed in his paper, though ably given by teachers of midwifery in general, would have been acceptable to him when first he entered on the fearful responsibility of his profession. How many more there are, who, after having gone through the usual course of lectures, might, in candour, join him in his assertion! Who is there, who, after leaving the obstetric school, and finding himself alone, for the first time, by the bed-side of a patient lingering through an anxious labour of doubtful issue, has not wished for some plainer, more concise, and positive, rules for his conduct, than his notes of the written lectures he has attended could supply his memory with?

In commenting upon, and adding to, the paper in question, I trust I shall be understood to be influenced by no other than the same motive which prompted its publication; namely, that of extending, by personal experience, the information respecting a subject of so much importance as the use of the midwifery forceps, and rendering it more available to those who as yet have not had the benefit of much practice.

It is asserted by L. G. that the forceps are only applicable in those cases in which there is a combination of the following circumstances:

1st. The safety of the woman must require delivery by art.
2d. The head must present.
3d. The labour must be sufficiently advanced.
4th. The dimensions of the pelvis must admit of their application, &c.

Now, however excellent these rules may be, they admit, in some respects, of farther addition; and, in others, of some slight correction. I shall endeavour to prove both these positions.

1. In the first place, it is not always necessary that the safety of the woman should, in addition to the other circumstances above enumerated, require the application of the forceps. We find their application often called for on account of the safety of the child, even where the labour, as far as the mother is concerned, promises to terminate without any great risk to her. Some such labour occurs, where the destruction of the child would be the inevitable consequence of neglecting to deliver with the forceps, because the mother's safety does not seem to require it. Many instances of still-born children are due to this very error. A long and arduous labour is left, in obedience to the Hunterian doctrine of relying on the powers of nature to the very last, to proceed and terminate without interference on our part, from an idea that the mother suffers but little from such a labour; but in the mean while the child dies: whereas, had mechanical assistance been given, after waiting a becoming time, the child would have been saved. L. G. therefore should have said, "the safety of the mother, *or that of the child*, must require delivery by art." This position may be thus exemplified. Mrs. B. became pregnant of her first child at the age of thirty-eight years. On the morning of Tuesday, the 18th of July last, she felt symptoms of approaching labour, when my attendance was required. Upon examination, the os uteri was found high, and dilated to about one inch in diameter. The membranous tumor was yet imperfectly formed; but the presentation of the head was ascertained. At eight in the evening a fresh examination was made, when the head was found to have descended considerably, and the os uteri to have dilated to the size of a crown-piece. The membranes ruptured at ten; the pains became strong and incessant; the vagina was filled with mucus, and the labour promised to be both quick and favourable. At the end of two hours more, very little, if any, progress had, however, been made. During a very sharp and lasting pain, the vertex of the head, which was now crowned by the thinned edges of the uterine circle, was found to fill the upper portion of the cavity of the sacrum; but it again receded at every intermission. At two in the morning the head appeared somewhat lower; not so much so, however, as might reasonably have been expected, from the incessant and very strong pains which the patient suffered. This led to a comparative examination at four o'clock, in order to ascertain the real progress of descent during the space of two hours; when it was found to have been so inconsiderable as scarcely to admit of admeasurement. At six in the morning things wore a somewhat different aspect. The head, being about one inch lower, appeared to mould itself to the passage with great difficulty; and the labour, notwithstanding the continuation of the pains and the great composure of the patient, threatened to be very lingering. In the mean while it was remarked, that the puckering of the scalp of the fetus, hitherto of the natural temperature of the surrounding parts, had lost much of its warmth, and felt chilly to the touch. The finger, on being passed round the head between it and the inner surface of the os uteri, could discover none of the lubrifying mucus; the uterine fibres felt rugose and on the stretch, as did those of the upper part of the vagina. Fears were entertained for the life of the child, if reliance were alone placed in the unabated and strong efforts of nature to mould its head within the cavity of an unyielding pelvis. A natural parturition, under such circumstances, to judge from the previous progress, would require a longer lapse of time spent in pains than the child could well be supposed to bear; and, although the mother's health and strength were such that no interference seemed necessary, yet what consolation would it have been to her to have given birth, after so much protracted suffering, *safely* and by the *unassisted efforts of nature*, to a still-born child! For who could doubt but that the child must soon have fallen a victim to the long duration of the labour? This then was the time for decision. The safety of the mother did not require the use of the forceps, but that of the child called for it immediately. Mrs. B. was therefore delivered with those instruments at nine o'clock in the morning of Wednesday; and, though the child could scarcely cry, and seemed at first inanimate, it lived, and ultimately did well.

2. The next point in L. G.'s paper is, "that the head must present in order to apply the forceps." It certainly cannot be denied, that on no other part of the fetus besides the head can those instruments be applied; but, by generalizing the *presentation* of the head in all cases of application of the forceps, has not the writer in question kept out of sight the possibility of the forceps being called for in cases of labours which began with a feet presentation, or after turning? To such cases there is not even the slightest allusion made in L. G.'s paper; so that I hope to be excused if I enter a little further into the subject: the which I do the more willingly, as none of the latest English writers on Midwifery appear to have noticed the application of the forceps under such circumstances.

There is scarcely an experienced practitioner who does not know that, in presentation of the extremities, and in cases where turning has been had recourse to, the head may be arrested in different parts of the pelvis; as for instance, above its abdominal brim, within its cavity or below its cavity, either in consequence of malposition or disproportion. If we attempt pulling by the shoulders, we run the risk of straining the vertebral ligaments of the neck. If we can reach the face with one hand, and endeavour to bring down the head by the introduction of one or two fingers within the mouth of the fetus, dislocation of the lower jaw may follow. If we study to correct the malposition of the head by imparting a rotatory motion to the body, we may give an unlucky twist to the neck. If we persist in completing the delivery by force, in spite of every obstacle, detruncation may ensue, and the accoucheur will be left in all the agonies of such an accident. Nor are these extraordinary or unlikely cases in the present day. The late Dr. Clarke, whose loss has not yet been supplied in the obstetrical profession, was once called to a case of detruncation; and, within the last six months, a similar accident happened to an old and experienced midwife, by whom I was, in consequence, called in for assistance. The possibility of the separation of the head from the trunk, is admitted on all hands, and mentioned even in all the modern treatises on Midwifery published in this country during the last twenty years; yet not one word is said of the mode of preventing such an accident,—the early application of the forceps after the extraction of the body; and much less is any direction given how to apply them in those cases. The same oversight has been committed by L. G.; and, to remedy this deficiency in his otherwise valuable paper, it will only be necessary to quote one out of the four cases mentioned by Smellie, in his Collections. For, although it would be an easy task to give more modern cases, taken from Gardien, Flaman, Ossiander, and Lobstein; Smellie may justly be preferred as a higher authority, and as being the first who devised this great improvement in the obstetric art.

"In the year 1755, I was called to a case in which the feet and hands of the child presented; the funis had not fallen down into the vagina; but, after the body was delivered, the head of the child stuck at the brim of the pelvis, on which I made several trials to bring it down into the vagina; but, finding the child was alive, by the pulsation of the arteries in the funis, I was afraid of overstraining the neck, if I repeated these trials and increased the force. The patient being in a supine position, I introduced a blade of the long forceps that were curved to one side, up along each side of the pelvis, while an assistant held up the body of the child, to give more room for their application; and, having fixed them on each side of the child's nose, while my right pulled the head with the instruments, and delivered it safely. These two successful cases [Smellie attended four such cases in the whole], gave me great hope that the above method would be of great service, and save the lives of many children, who *are generally lost by overstraining the neck in delivering the head*," &c.

Another case, of a more recent date, will illustrate this part of my remarks still further, as well as supply us with clearer directions for using the forceps under such circumstances. A woman, aged twenty-two years, healthy, and pregnant with her first child, was taken in labour late at night. The membranes gave way almost immediately afterwards; when, on examination, the fetus was found to lie across the pelvis, the face resting on the left iliac excavation, and the abdomen presenting. Some attempts were made to correct this position by endeavouring to raise high within the womb the lower extremities, so as to enable the head to slide down. These proved fruitless, and turning was ultimately had recourse to, by which the head was brought into the cavity of the pelvis. During this operation care was taken to keep the face within the hollow of the sacrum; but once so placed, considerable resistance was experienced, and no further progress could be made, in spite of every reasonable and prudent effort by pulling. The fetus now seemed convulsed, and the vibrations of the chord became more faint. The forceps were instantly had recourse to. The fetus being wrapt round with hot napkins, was committed to the charge of a nurse, and directed towards the abdomen of the mother. One of the branches was introduced, and applied over the right ear of the fetus, the second over the left, with great facility. The blades being locked without the vagina, the head was first gently raised, then gradually depressed, so as to bring the chin nearer to the chest; a mixed lateral and descending motion was imparted to the forceps; when, after a few minutes, the patient was readily delivered of a fetus of usual dimensions and weight, which required some care before it gave signs of life, but which ultimately lived. The forceps in such cases must be of longer dimensions than those commonly in use, in order to admit of more space for the handles, which, after the introduction of the blades, ought to form an obtuse angle with the body of the child.

3. With respect to the third circumstance mentioned by L. G. namely, "that the labour must be sufficiently advanced before the forceps are applied," I have only a few words to offer. It was once a received rule,* that the head of the child should have rested on the perineum for six hours before we thought of applying the forceps ; and almost every lecturer in London is in the habit of repeating another rule, equally general, "that the forceps may be applied when an ear of the child can be felt from the os externum." Both rules are bad, as far as they are intended to be exclusive. If we are called to a case in which the use of the forceps is required, and we find the head resting on the perineum, or that an ear can be felt, so much the more easy will be the application of the instrument. Or, should we have suffered the labour under our immediate care to proceed so far without interference, as that the head rests on the perineum, or an ear can be felt, and next think it necessary to use the forceps, the same observation will hold good with regard to the easy facility of their application. But I contend, from experience, that, to limit the propriety of applying the forceps, as to time, to these two circumstances alone, is contrary to the best interests of both the patient and the practitioner, and inimical to the advancement of our art. Were I to suggest a general rule for the satisfaction of young practitioners in all such cases, I should say, "whenever the uterine circle is obliterated, or its dilation is such that the head is likely to emerge from it without lacerating it, and the labour has in every other respect proceeded in such a manner as to call for the forceps, apply them forthwith with care and caution, whether you can feel an ear or not, whether the head of the fetus rests or not as low as the perineum." The higher the head, the longer must of course be the forceps. To limit ourselves to what are commonly called Denman's or Clarke's forceps, measuring barely twelve inches in length, is ridiculous: for, if we admit the correctness of Dr. Douglas's assumption,† "that the cervix uteri and vagina occupy just five-tenths of the whole length of the canal interested in the act of parturition;" and if we reflect, moreover, that full five inches of the small forceps will be necessarily left without the labia for the grasp of the operator, the impracticability of delivering women with such forceps will become obvious; unless, indeed, the head be within an iota of its final expulsion at the time of the application. It is a serious error to suppose, that the longer the forceps the greater the probability of mischief. Of the numerous instances of application of the French forceps that I have witnessed abroad, in none have I seen lacerations of any

* See Denman's Introduction to the Practice of Midwifery, page 390, fifth edition.

† See a paper by Dr. Douglas, of Dublin, inserted in the sixth volume of the Medical Transactions of the London College of Physicians, lately published, page 386.

kind take place, and in most of them the child was saved. Within the last three months, indeed within the last week, as some of my readers may perhaps recollect, it has fallen to my lot to witness the dreadful effects of *short forceps* clumsily handled, in the case of a poor woman, now on the books of the Westminster General Dispensary, suffering agonies some weeks after an instrumental labour, during which a *partial* laceration of the os uteri, a *total* laceration of the perineum, and a perforation of the bladder producing *stillicidium*, took place. I would not by any means recommend such a preposterous instrument as the French forceps to the attention of the English practitioner; but I would have him adopt that size which he can best manage for the relief of his patient and the safety of the child, and suitable to the nature of the case; nor would I wish him to be the slave of any general rule, other than what is derived from practical experience and *personal* observation.

4. On the subject of the fourth circumstance mentioned by L. G. that "the pelvis must be of sufficient dimensions to admit the passage of the head without an evacuation of its contents," there cannot be a dissentient voice among practitioners. But I may be allowed to point out a contradiction into which L. G. has fallen in the course of his remarks on this part of his subject; and I am sure he will take it in good part that I should do so; as it is with a view to prevent his readers from feeling perplexed in a matter of so much importance. L. G. says, "where the passage of the entire head is mechanically impossible, the forceps are, of course, totally inapplicable; but, where it is calculated that the aperture of the pelvis, although contracted, is equal to a space of from *two inches and a half* to three inches, *I believe delivery by the forceps to be frequently practicable.*" Again, a few lines farther, he states that "in those cases of contracted pelvis in which the forceps are successful, they do not act by compressing the head; for the diameter of the forceps, from blade to blade, will always be *more than three inches*, &c." Surely then delivery by the forceps, in cases of contracted pelvis to the degree mentioned before, cannot be *practicable*. How, we would ask, can a diameter of *more than three inches* be made to go through an aperture of *two inches and a half*, or even *three* inches? The fact is, that when there is reason to suspect that the aperture of the pelvis is contracted to less than three inches, we employ forceps, the greatest distance of whose blades is *two inches and a half*, with a view to diminish, by an equal pressure on the lateral regions of the head, its natural transverse diameter.

The *directions* which follow in L. G.'s paper, for the right application of the forceps, are so minute, clear, and accurate, that I am sure those of his readers who are yet young in the practice of midwifery must feel highly indebted to him: nor do I conceive that any instructions on this subject, contained in any English work on midwifery, can be considered as doing away the necessity of those *directions*, as the author very modestly supposes. On such a subject we cannot be too minute; and some of our best masters, Denman in particular, are purposely too short and superficial. There are a few points in L. G.'s instructions in which some difference of opinion might arise; but this seems not a fit opportunity for stating them.

I shall conclude with a few words on the choice of the forceps to be employed. This must depend greatly on the nature of the case under our management. Dr. Haighton's forceps are decidedly preferable in most cases, provided that care be taken to have them made less thick in the outer edges than they are generally made. We would otherwise be wasting a space of nearly two-tenths of an inch, without any good purpose; for stoutness of the instrument is not required. In cases where the head of the child has been suffered to descend as far as the perineum, what are called Denman's or Clarke's forceps are good enough: indeed, in such cases, a single blade of any forceps, and your finger, will most probably accomplish the whole thing, with certainly less probability of mischief to the mother or child. Somewhat longer forceps than Haighton's will be found necessary where their application is called for above the abdominal brim of the pelvis. Such cases will occur, and are managed with less difficulty than one would be disposed to believe *à priori.* Assalini's forceps are very handy, and, in the hands of a young practitioner, less likely to do harm, because easier of application, and requiring no cross-locking of the blades. The latter forceps give, besides, a greater command over the fetus to the operator than any other. Where the person who is to employ the forceps is accustomed to bear in mind the two axes of the pelvis, the straight-blade forceps are the most preferable; for here we have not a right and a left blade to puzzle the beginner, and to call away his attention from more important objects, during the application of the instrument.

There is a common defect in all the short forceps which I have seen employed and recommended by lecturers, that should be noticed; namely, that when locked, and the handles brought in close contact, an open space of seven-tenths of an inch, or thereabout, is left between the upper extremities of the blades. By this we expose ourselves to the following great inconvenience: suppose such forceps be made to pass, during the extraction of the head, through a diameter less than the greatest distance between the blades, the compression excited by the surrounding bones of the pelvis on the blades of the forceps will inevitably cause their upper extremities to approach nearer, and perhaps to come wholly in contact: nor would this, indeed, be a bad result, as it would certainly tend to diminish the diameter of the head in proportion. But we are directed to *fasten a napkin or tape* tightly round the handles of the forceps; and what then? Why, no such effect as that above-described can of course take place; for the approaching of the upper extremities of the blades of the forceps by dint of pressure external to them, implies a corresponding separation of the handles below their junction, which separation the napkin or tape will effectually prevent. Hence the pretended improvement of this very space between the extremities of the blades becomes a direct obstacle to delivery; since, if the aperture of the pelvis be such, that, in endeavouring to extract the child, the blades must come in contact at their extremities, and this effect be rendered impossible by the mechanical resistance of the ligature round the handles, a total check will be given to the completion of the labour; unless, by downright violence, we should succeed in tearing every surrounding part, as a contemporary writer has expressed it, "into one hideous gap."

8, *Saville Row, Sept.* 10, 1820.

